# Survey on the Occurrence of Zoonotic Bacterial Pathogens in the Feces of Wolves (*Canis lupus italicus*) Collected in a Protected Area in Central Italy

**DOI:** 10.3390/microorganisms12112367

**Published:** 2024-11-20

**Authors:** Fabrizio Bertelloni, Giulia Cagnoli, Valentina Virginia Ebani

**Affiliations:** 1 Department of Veterinary Science, University of Pisa, Viale delle Piagge 2, 56124 Pisa, Italy; fabrizio.bertelloni@unipi.it (F.B.); giulia.cagnoli@vet.unipi.it (G.C.); 2 Centre for Climate Change Impact, University of Pisa, 56124 Pisa, Italy

**Keywords:** Italian wolf (*Canis lupus italicus*), zoonosis, *Yersinia enterocolitica*, *Listeria monocytogenes*, Shiga Toxin-Producing *Escherichia coli* (STEC), antimicrobial resistance

## Abstract

Previous investigations have explored the involvement of wolves in parasitic and viral diseases, but data on the zoonotic bacteria are limited. The aim of this study was to assess the occurrence of bacterial zoonotic agents in 16 wolf (*Canis lupus italicus*) fecal samples collected in a protected area in Central Italy. *Campylobacter* spp., *Salmonella* spp., *Yersinia* spp., *Listeria monocytogenes,* and Shiga Toxin-Producing *Escherichia coli* (STEC) were investigated by culture, while polymerase chain reaction (PCR) was employed to detect *Coxiella burnetii*, *Mycobacterium* spp., *Brucella* spp., and *Francisella tularensis*. The presence of Extended Spectrum β-Lactamase (ESBL)- and carbapenemase-producing Enterobacteriaceae was also evaluated, using selective isolation media and detection of antimicrobial resistance genes. All samples were negative for *Campylobacter* spp., *Salmonella* spp., *C. burnetii*, *Mycobacterium* spp., *Brucella* spp., *F. tularensis*, and carbapenemase-producing Enterobacteriaceae. One sample tested positive for *Yersinia aldovae* and three for *Yersinia enterocolitica* BT1A. One *L. monocytogenes* (serogroup IIa) and one STEC, carrying the *stx1* gene, were isolated. Two ESBL isolates were detected: one *Serratia fonticola*, carrying *bla_FONA-3/6_* gene, and one *Escherichia coli*, carrying *bla_CTX-M-1_* gene. Both ESBL isolates were resistant to different antimicrobials and therefore classified as multi-drug-resistant. Our data suggest that wolves are potential carriers of zoonotic bacteria and may contribute to the environmental contamination through their feces.

## 1. Introduction

The wolf (*Canis lupus*) is an adaptable wild animal species, distributed in many parts of the world [1]. It was the first domesticated species, but over the centuries, wolves have posed a constant problem for human populations [2]. In Western Europe, in particular in Central Italy and Spain, by the 1970s, only few individuals survived [3]. Since then, conservation and reintroduction programs have been established, leading to a constant growth in the wolf population [4]. Recent data show the wide spread of this species in many parts of Italy, with recolonization of several habitats [4]. Currently, the Italian wolf (*Canis lupus italicus*) is a protected species in Italy [5], with an estimated population of about 3300 individuals [6].

Wolves are generalist apex predators, mainly preying on wild ungulates [7]; therefore, it is plausible that wolves could be exposed to various pathogens and undergo an accumulation process [8,9,10]. Many recent studies conducted in Italy support this hypothesis. In particular, most of these surveys were focused on parasites [8,11,12,13,14,15,16,17,18,19,20] and viral agents [12,21,22,23,24,25,26,27,28].

Conversely, only few studies have been carried out to investigate the presence of bacterial pathogens in the feces of these animals [29,30,31]. Wolves’ feces may contaminate the environment and become sources of pathogens for other animals contributing to the amplification of the epidemiological scenario of given agents. This amplification can become a public health issue considering that most of them may cause zoonoses.

In view of the scarce data on bacterial infections in wolves, the aim of the present survey was to investigate the occurrence of the most important zoonotic bacterial pathogens in feces, collected from the environment, of wolves living in a national park located in Central Italy. For this purpose, *Listeria monocytogenes*, *Campylobacter* spp., *Salmonella* spp., *Yersinia* spp., Shiga Toxin-Producing *Escherichia coli* (STEC), Extended Spectrum β-Lactamase (ESBL)-producing and carbapenemase-producing Enterobacteriaceae were investigated using traditional bacteriological examinations. In addition, presence of *Coxiella burnetii*, *Mycobacterium* spp., *Brucella* spp., and *Francisella tularensis* was assessed by molecular investigations.

## 2. Materials and Methods

### 2.1. Study Area

The study was conducted in the Foreste Casentinesi National Park, located in the Northern Italian Apennines, between the Tuscany and Emilia-Romagna regions, and covering an area of about 36,000 hectares of woodland. The park is the home of many animal species, such as wild ungulates, small mammals, wild birds, amphibians, and reptiles. About 100 wolves have been estimated to be present throughout the park [32]. The park is a protected area with decreased anthropic pressure. However, medium-size towns and small villages are present, as well as some extensive or semi-extensive breeding of small ruminants, bovine, and swine. The park is frequently visited by tourists for recreational activities.

### 2.2. Sampling

Samples were collected for 14 consecutive days in April 2022. Only intact feces (with an evident mucous layer on the surface, still soft, not covered with dust and/or molds), identified as wolf scat on the basis of their shape, size, and smell [33], were taken; about 10–50 g of feces was collected in sterile 300 mL jars and stored in refrigerated condition. The samples were sent to the Laboratory of Infectious Diseases of the Department of Veterinary Science, University of Pisa, and analyzed within 24 h from sampling. Since the collected samples could not be attributed to known animals, it cannot be excluded that they came from the same wolves.

### 2.3. Bacteriological Analyses

Each fecal sample was divided into five smaller portions, in a biosafety cabinet and using sterile instruments. Each aliquot contained approximately from 1 to 10 g of feces, depending on the total available amount of the samples. Four aliquots were used for enrichment cultures to isolate specific pathogens, namely *L. monocytogenes*, *Campylobacter* spp., *Yersinia* spp., *Salmonella* spp., Shiga Toxin-Producing *Escherichia coli* (STEC), Extended Spectrum β-Lactamase (ESBL)-producing and carbapenemase-producing Enterobacteriaceae. All obtained isolates were stored at −80 °C in Brain Heart Infusion (BHI, Oxoid Ltd., Basingstoke, UK) by the addition of 20% glycerol as cryoprotectant for successive analyses.

The remaining aliquot was used in molecular investigations to detect *Coxiella burnetii*, *Mycobacterium* spp., *Brucella* spp., and *Francisella tularensis.*

All polymerase chain reaction (PCR) assays described below were performed using the EconoTaq PLUS 2x Master Mix (Lucigen Corporation, Middleton, WI, USA) and the automated thermal cycler SimpliAmp™ Thermal Cycler (Applied Biosystems, Waltham, MA, USA). The PCR products were analyzed by electrophoresis on 1.5% agarose gel stained with Ethidium bromide. A 100 bp DNA Ladder Ready to Load (Solis BioDyne, Tartu, Estonia) was used as a DNA marker.

#### 2.3.1. Bacteriological Cultures

### Listeria monocytogenes

Feces were diluted 1:10 in ONE Broth-Listeria (Oxoid Ltd.) and, after mixing by stomacher, incubated at 30 °C for 24 h. Then, one loopful from each culture was stretched on Agar Listeria Ottaviani Agosti (ALOA) (Biolife, Milan, Italy) and incubated at 37 °C for 48 h. Suspected colonies were subcultured on Tryptic Soy Agar (TSA) (Biolife) and initially confirmed as *Listeria* spp. and suspected *L. monocytogenes* by Gram staining, catalase, oxidase, CAMP test with *Staphylococcus aureus* (ATCC 25923) and *Rhodococcus equi* (ATCC 6939), and acid production from mannitol, rhamnose and xylose. After DNA extraction from an overnight culture using a commercial kit, DNA Plus Kits (Zymo Research, Irvine, CA, USA) and following the manufacturer’s instructions, presumptive *L. monocytogenes* isolates were confirmed by PCR assays searching for the genes *prs* and *prfA*, as previously described [34,35]. Moreover, PCR assays were carried out to type the obtained *L. monocytogenes* isolate, searching for the genes *lmo1118*, *ORF2819*, *ORF2110*, and *flaA* that allow to identify the serogroup [34,36].

### Campylobacter spp.

Feces were diluted 1:10 in Bolton Selective Enrichment Broth (Bolton Broth, plus Bolton Broth Selective Supplement and Laked Horse Blood) (Oxoid Ltd.), mixed by stomacher and incubated in microaerobic environment (5% oxygen, 10% CO_2_), for 4 h at 37 °C, and subsequently for 44 h at 42 °C. Then, one loopful was subcultured on Campylobacter Blood-Free Selective Medium (formally “modified Charcoal Cefoperazone Deoxycholate Agar, mCCDA) (Oxoid Ltd.) and incubated for 48 h at 42 °C in microaerobic environment (5% oxygen, 10% CO_2_). Specific PCR targeting the gene 23SrRNA was carried out to confirm the genus *Campylobacter* for suspected colonies [37].

### Yersinia spp.

Feces were diluted 1:10 in Peptone Sorbitol Bile (PSB) broth (Biolife), mixed by stomacher and incubated at 4 °C for 21 days. Subsequently, one loopful from each sample was stretched on Cefsulodin Irgasan Novobiocin (CIN) agar (Biolife) and incubated at 30 °C for 24 h. Suspected colonies were screened with Triple Sugar Iron (TSI) agar (Biolife) and for urease activity with Urea Broth (Oxoid Ltd.); *Yersinia* species were identified with API20E (Biomerieux, Marcy l’Etoile, France). *Yersinia enterocolitica* isolates were successively characterized on the basis of biochemical tests to distinguish the biotype, as previously described [38]. The isolates were analyzed with singular PCR assays to detect the following virulence genes: *ail*, *virF*, *ystA*, *ystB* and *inv*. DNA analyzed in PCR was extracted from overnight culture with a commercial kit, DNA Plus Kits (Zymo Research, Irvine, CA, USA), following manufacturer’s instructions; primers and PCR protocols previously reported by other authors were adopted [39,40,41].

### Salmonella spp.

Feces were diluted 1:10 in Buffered Peptone Water (BPW) (Biolife) and, after mixing by stomacher, incubated at 37 °C for 24 h. Subsequently, 1 and 0.1 mL from BPW were subcultured in Selenite Broth (Biolife) and Rappaport Vassiliadis Broth (Biolife), respectively; Selenite Broth was incubated at 37 °C for 24 h, whereas Rappaport Vassiliadis Broth was incubated at 41.5 °C for 24 h. After incubation, 1 loopful from each broth was cultured on both Salmonella Shigella (SS) agar (Biolife) and Brilliant Green Agar (BGA) (Biolife) and incubated at 37 °C for 24 h. Suspected colonies were evaluated with conventional biochemical tests (TSI, urease, o-nitrofenil-β-D-galattopiranoside (ONPG), indole, Voges-Proskauer (VP), Lisyne decarboxylases and malonate) and confirmed as *Salmonella* spp. by PCR detection of *invA* gene [42].

### Shiga Toxin-Producing Escherichia coli (STEC)

One loopful from BPW, after incubation, was cultured on Tryptone Bile X-glucuronide (TBX) agar (Biolife) and incubated at 42 °C for 24 h. Three distinct colonies were selected and subcultured on Tryptone Soy Agar (TSA) (Biolife) to be confirmed as *E. coli* by Api20E (Biomerieux). Identified isolates were tested for the presence of the genes *stx1* and *stx2*, coding Shiga-toxin 1 and Shiga-toxin 2, respectively, with PCR, using primers and protocol previously described [43].

### Extended Spectrum β-Lactamase- and Carbapenemase-Producing Enterobacteriaceae

After incubation in BPW as previously described, one loopful from each sample was stretched on both ChromArt ESBL AGAR (Biolife) and ChromArt CRE AGAR (Biolife); both media were incubated at 37 °C for 48 h. From each medium, 1 to 3 colonies different in color, size and morphology were subcultured on Violet Red Bile Glucose Agar (VRBGA) (Biolife), and glucose fermenting isolates were tested for oxidase production. Oxidase negative isolates were further identified at species level with API20E (Biomeriux).

Enterobacteriaceae isolated on ChromArt ESBL AGAR were tested for the presence of the antimicrobial resistance genes *bla_TEM_*, *bla_SHV_*, *bla_CTX-M_*, whereas Enterobacteriaceae isolated on ChromArt CRE AGAR were tested to detect the antimicrobial resistance genes *bla_NDM_*, *bla_KPC_*, *bla_OXA-48_*, *bla_IMP_*, *bla_VIM_*. After DNA extraction from overnight cultures using the commercial kit, DNA Plus Kits (Zymo Research, Irvine, CA, USA), PCR assays were carried out using primers and protocols previously described by other authors [44,45,46].

The positive PCR products were sequenced (BMR Genomics, Padova, Italy), and the obtained sequences were compared with a gene bank database using Basic Local Alignment Search Tool (BLAST) and FASTA (https://www.ebi.ac.uk/Tools/sss/fasta/, accessed on 30 September 2023).

All primers and conditions of the PCR assays employed to characterize the bacterial isolates are reported in Table 1.

#### 2.3.2. Antimicrobial Susceptibility Test

All obtained bacterial isolates were submitted to the disc diffusion test to evaluate their antimicrobial susceptibility, following the line guide by Clinical Laboratory Standard Institute (CLSI) [47]. The following antimicrobials (Oxoid) were tested for *L*. *monocytogenes*: ampicillin (2 μg), meropenem (10 μg), erythromycin (5 μg), and trimethoprim-sulfamethoxazole (1.25/23.75 μg); the results were interpreted according to European Committee on Antimicrobial Susceptibility Testing (EUCAST) [48]. The isolates belonging to Enterobacteriaceae were tested with the following antimicrobials (Oxoid): ampicillin (10 μg), amoxicillin-clavulonate (20/10 μg), cefoxitin (30 μg), cefotaxime (30 μg), ceftiofur (30 μg), imipenem (10 μg), ertapenem (10 μg), aztreonam (30 μg), chloramphenicol (30 μg), tetracycline (30 μg), enrofloxacin (5 μg), ciprofloxacin (5 μg), gentamicin (10 μg), amikacin (30 μg), and trimethoprim-sulfamethoxazole (1.25/23.75 μg); the results were read according to CLSI [49]. *Yersinia* spp. isolates were tested with the same antimicrobials used for Enterobacteriaceae but not against ampicillin and amoxicillin-clavulonate, due to their intrinsic resistance.

#### 2.3.3. Molecular Detection of Pathogens

DNA was extracted from approximately 150 mg of each fecal sample using a commercial kit, Quick-DNA Fecal/Soil Microbe Miniprep Kit (Zymo Research, Irvine, CA, USA), following the manufacturer’s instructions, and stored at −20 °C.

To detect a 687 bp portion of the gene *IS1111a* of *C. burnetii*, the primers Trans-1 and Trans-2, along with the protocol proposed by Berri et al. [50], were utilized.

The presence of bacteria belonging to the *Mycobacterium* genus was assessed using the primers MycogenF and MycogenR, which allowed for the amplification of 1030 bp portion of the 16SrDNA [51].

In order to detect *Brucella* spp. DNA, a PCR with the primers B4 and B5, targeting a 223 bp fragment of the gene *bcsp31* was performed [52].

Lastly, the presence of *F. tularensis* was evaluated using the primers TUL4–435 and TUL4–863, which allowed the amplification of a 400 bp fragment of the gene *TUL4* [53].

Primers and PCR conditions of the employed protocols are reported in Table 2.

## 3. Results

A total of 16 distinct wolf fecal samples were collected and analyzed. Of these, 7 (43.75%; 95% CI: 19.44.00–68.06%) samples were positive for at least one of the investigated bacteria, and among them, 1/16 (6.25%) sample was positive to two different pathogens (*L. monocytogenes* and *Y. enterocolitica*) (Table 3).

No samples were positive for *Salmonella* spp., *Campylobacter* spp., and carbapenemases-producing Enterobacteriaceae. One strain of *L. monocytogenes* was isolated (sample 1), and the subsequent molecular characterization revealed that it belonged to the IIa serogroup having the genes *lmo0737* and *flaA*.

Four *Yersinia* spp. isolates were obtained: one *Yersinia aldovae* (sample 2) and three *Yersinia enterocolitica* Biotype 1A (samples 1, 6, 7). PCR detected no virulence genes in *Y. enterocolitica* isolates.

A total of 48 *E. coli* strains were isolated, three from each fecal sample; among them, one strain (sample 12) tested positive for the gene *stx1*, categorizing it as belonging to the pathotype STEC.

*Listeria monocytogenes* and all *Yersinia* spp. and *E. coli* isolates were susceptible to the tested antimicrobials.

Two ESBL isolates were detected and characterized. The first isolate (sample 13) was classified as *Serratia fonticola*, and it tested positive for *bla_CTX-M_*. The PCR product showed a 87.37% identity with the *bla_FONA-3/6_* gene. The second isolate (sample 16) was classified as *E. coli* and tested positive for *bla_CTX-M_*. Sequence analyses showed a 100% identity with *bla_CTX-M-1_* gene. The ESBL *S. fonticola* isolate was resistant to ampicillin and amoxicillin-clavulonate (penicillins class), cefoxitin, cefotaxime, and ceftiofur (cephalosporins class), and aztreonam (monobactams class), whereas the ESBL *E. coli* strain was resistant to ampicillin (penicillins class), cefoxitin, cefotaxime, and ceftiofur (cephalosporins class), aztreonam (monobactams class), and tetracycline (tetracyclines class). Both isolates can be classified as multi-drug-resistant (MDR) [54].

The DNA of *C. burnetii*, *Mycobacterium* spp., *Brucella* spp., and *F. tularensis* was not detected in any of the analyzed samples.

## 4. Discussion

In recent years, there has been a growth in the number of wolves on Italian territory; wolves are increasingly present in forest areas, but not rarely they are sighted even near inhabited centers. Considering the difficulty in collecting biological samples from these animals, the role of wolves in the epidemiology of some pathogens has not been fully elucidated. In particular, studies have been carried out on parasitic and viral agents, but little information on bacterial pathogens has been obtained. Bacteria belonging to different species may be excreted in feces becoming responsible for environmental contamination. Wolf feces on forest areas’ soil may be a direct source of infection for other animals, such as small mammals, birds, reptiles, amphibians, insects, but also hunting dogs, wild boars, and wild ruminants. In addition, mainly after abundant rainfall, fecal material can run off, amplifying the concern of microbial dissemination.

The present investigation did not detect relevant pathogens, such as *Campylobacter* spp., *Salmonella* spp., *F. tularensis*, *C. burnetii*, *Brucella* spp., and *Mycobacterium* spp. These findings may be attributed to the low circulation of the agents in the sampling area, despite the absence of related studies in the existing literature. Moreover, the fecal shedding of these bacteria usually is discontinuous, mainly in clinically healthy animals, and as other routes of excretion are possible; other biological samples should be investigated. Wild animals can be carriers, usually asymptomatic, of these pathogens [55,56,57,58,59,60], and infections in wolves have been reported for *Salmonella* spp. [61], *F. tularensis* [62], *Brucella* spp. [63], *Mycobacterium bovis* [64], and *Mycobacterium caprae* [65].

On the other hand, our investigation found zoonotic bacteria, such as *Y. enterocolitica* and *L. monocytogenes*, less investigated in wildlife, but able to infect other animals.

*Listeria monocytogenes* causes severe human infection characterized by a relevant proportion of hospitalized patients and mortality [66]. Pigs and cattle are the main reservoirs among domestic animals [66,67], but wild animals can harbor the pathogen as well. The role of wildlife in the epidemiological cycle of *L. monocytogenes* remains a topic of ongoing investigation [67], and there are no data in the available literature regarding the occurrence of *L. monocytogenes* in wolves. The strain cultured in our survey belongs to the serogroup IIa, which includes serotypes 1/2a and 3a, frequently found in human and animal infections [68].

*Yersinia enterocolitica,* isolated from 18.75% of the analyzed samples, is confirmed to be present in wildlife, even though non-virulent strains are the most frequently detected [69,70,71,72,73]. There are no available data on wolves as carriers of *Yersinia* spp. in the literature; therefore, our results cannot be compared to other epidemiological situations. Three isolates of our study were classified as *Y. enterocolitica* Biotype 1A; this is a non-virulent biotype previously found in wild animals and environment [74]. However, the virulence potential of *Y. enterocolitica* Biotype 1A was recently re-evaluated, because it has been associated with cases of human infections [41,75]. The isolation of *Y. aldovae*, species not related to human or animal infections, is not surprising because it is commonly isolated from aquatic ecosystems and soil [76].

The detection of one STEC strain is interesting because, to the best of our knowledge, this is the first report of this pathotype in wolves. STEC are bacteria responsible for severe human diseases, such as hemorrhagic colitis in adults and hemolytic uremic syndrome in children [77]. Bovine are identified as the main reservoirs [78], although wildlife, mammals and birds, have been proven to harbor this *E. coli* pathotype [77,79,80].

Two strains, one *E. coli* and one *S. fonticola*, resulted as ESBL-producing bacteria with *bla_CTX-M-1_* and *bla_FONA_* genes, respectively. Gonçalves and colleagues (2012) registered a prevalence of 5.5% ESBL-producing *E. coli* in fecal samples of Iberian wolves (*Canis lupus signatus*) in Portugal and detected some relative resistance genes, including the gene *bla_CTX-M-1_*, which is known as one of the most commonly detected ESBL genes in Europe [9]. *Serratia fonticola* is an environmental bacterium rarely associated with human infections [81] and intrinsically resistant to penicillins and cephalosporins due to the presence in the chromosome of the ESBL resistance gene *bla_FONA_* [82,83]. The detection of two (12.5%) ESBL-producing Enterobacteriaceae strains in a small number of analyzed samples shows that these bacteria are circulating, as also highlighted by previous studies that found these microorganisms in humans and domestic and wild animals [84,85]. Currently, the spreading of ESBL- and carbapenemase-producing bacteria represent a relevant concern because Extended Spectrum beta-lactams and carbapenems are antimicrobials commonly used in human medicine, mainly for the treatment of highly drug-resistant bacteria [86,87]. No carbapenemase-producing Enterobacteriaceae were detected in the present investigation. This finding is quite in agreement with previous surveys that found low percentage of carbapenem-resistant bacteria in wildlife [88]. Conversely, some studies have reported high rate of detection in wildlife, especially in seagulls (15.9% in Australia and 19.4% in France), and wild boars (0.6–13.4% in Algeria) [89].

Antimicrobial resistance was found only in the two ESBL strains. These isolates were resistant to antimicrobials belonging to different classes, and therefore, they were classified as MDR. This finding showed that a single bacterial strain can harbor resistance to several antimicrobials [90] and suggested that animals, such as wild species, never treated with antibiotics, may acquire resistant bacteria from the environment.

## 5. Conclusions

The small number of samples that it was possible to collect does not allow for clarification of the role of wolves in the epidemiological cycles of the investigated pathogens. The negative results could be related to the absence of the searched pathogens in the sampling area and do not exclude wolves as susceptible animals. On the other hand, the detection of bacteria, such as *L. monocytogenes*, *Y. enterocolitica*, STEC, and ESBL-producing *E. coli*, suggests that wolves can be infected by them and consequently contribute to their spreading in the environment. Moreover, the detection of MDR bacteria and of bacteria harboring resistance genes showed that these animals may contribute to the antimicrobial resistance issue. The examined fecal samples were collected directly from the ground where wolves had placed them; therefore, the detection of antimicrobial-resistant bacteria highlights the importance of the environmental contamination. In fact, the finding of these zoonotic bacteria does not suggest wolves as a direct source of infection for humans, but shows that the fecal contamination contributes to the dissemination of agents which could indirectly infect people.

The present study, although carried out on a small number of samples, is the first focused on a large number of zoonotic bacterial pathogens; the results are preliminary but suggest, also from a One Health perspective, monitoring wolf populations in order to increase the knowledge about the bacterial circulation in wildlife and their role in the environmental contamination. In addition, metagenomic analyses could be useful to provide unbiased detection of all the pathogenic and nonpathogenic microflora present in the analyzed samples.

## Figures and Tables

**Table 1:** Primers and related information for the PCR assays employed to characterize the bacterial strains isolated from fecal samples.

Application	Gene	Primers	Sequences	Amplicons (bp)	Annealing Temperature °C	Ref.
*Listeria monocytogenes* confirmation and typing	*prs*	PRS_1	GCTGAAGAGATTGCGAAAGAAG	370	53	[34]
		PRS_2	CAAAGAAACCTTGGATTTGCGG			
	*prfA*	LIP1	GATACAGAAACATCGGTTGGC	274	53	[35]
		LIP2a	GTGTAATCTTGATGCCATCAGG			
	*lmo0737*	LMO0737_1	AGGGCTTCAAGGACTTACCC	691	53	[34]
		LMO0737_2	ACGATTTCTGCTTGCCATTC			
	*lmo1118*	LMO1118_1	AGGGGTCTTAAATCCTGGA	906		
		LMO1118_2	CGGCTTGTTCGGCATACTTA			
	*ORF2819*	ORF2819_1	AGCAAAATGCCAAAACTCGT	471		
		ORF2819_2	CATCACTAAAGCCTCCCATTG			
	*ORF2110*	ORF2110_1	AGTGGACAATTGATTGGTGAA	597		
		ORF2110_2	CATCCATCCCTTACTTTGGAC			
	*flaA*	FlaA-F	TTACTAGATCAAACTGCTCC	538	61	[36]
		FlaA-R	AAGAAAAGCCCCTCGTCC			
*Campylobacter* spp. confirmation	23S rRNA	23SF	TATACCGGTAAGGAGTGCTGGAG	650	59	[37]
		23SR	ATCAATTAACCTTCGAGCACCG			
*Yersinia enterocolitica* virulence genes detection	*ail*	9A	GTTTATCAATTGCGTCTGTTAATGTGTACG	454	60	[40]
		10A	CTATCGAGTTTGGAGTATTCATATGAAGCG			
	*virF*	11A	AAGGTTGTTGAGCATTCACAAGATGG	700		
		12A	TTTGAGTGAAATAAGACTGACTCGAGAACC			
	*inv*	invF	TGCCTTGGTATGACTCTGCTTCA	1114	63	[41]
		invR	AGCGCACCATTACTGGTGGTTAT			
	*ystA*	ystAF	ATCGACACCAATAACCGCTGAG	79	61	[39]
		ystAR	CCAATCACTACTGACTTCGGCT			
	*ystB*	ystBF	GTACATTAGGCCAAGAGACG	146		
		ystBR	GCAACATACCTCACAACACC			
*Salmonella* spp. confirmation	*invA*	invAF	GTTGTACCGTGGCATGTCTG	930	50	[42]
		invAR	GCCATGGTATGGATTTGTCC			
Shiga Toxin-Producing *Escherichia coli* (STEC) detection	*stx1*	stx1F	ATAAATCGCCATTCGTTGACTAC	180	60	[43]
		stx1R	GAACGCCCACTGAGATCATC			
	*stx2*	stx2F	GGCACTGTCTGAAACTGCTCC	255		
		stx2R	TCGCCAGTTATCTGACATTCTG			
Extended Spectrum β-Lactamase genes detection	*bla_TEM_*	blaTEMF	GCACGAGTGGGTTACATCGA	310	60	[45]
		blaTEMR	GGTCCTCCGATCGTTGTCAG			
	*bla_SHV_*	SHV-F	TTCGCCTGTGTATTATCTCCCTG	854	50	[44]
		SHV-R	TTAGCGTTGCCAGTGYTCG			
	*bla_CTX-M_*	CTX-F	ATGTGCAGYACCAGTAARGTKATGGC	593	60	
		CTX-R	TGGGTRAARTARGTSACCAGAAYCAGCGG			
Carbapenemase genes detection	*bla_NDM_*	NDM-F	GGTTTGGCGATCTGGTTTTC	621	52	[46]
		NDM-R	CGGAATGGCTCATCACGATC			
	*bla_KPC_*	KPC-F	CGTCTAGTTCTGCTGTCTTG	798		
		KPC-R	CTTGTCATCCTTGTTAGGCG			
	*bla_OXA-48_*	OXA-F	GCGTGGTTAAGGATGAACAC	438		
		OXA-R	CATCAAGTTCAACCCAACCG			
	*bla_IMP_*	IMP-F	GGAATAGAGTGGCTTAAYTCTC	232		
		IMP-R	GGTTTAAYAAAACAACCACC			
	*bla_VIM_*	VIM-F	GATGGTGTTTGGTCGCATA	390		
		VIM-R	CGAATGCGCAGCACCAG			

**Table 2:** Primers and related information for the PCR assays employed for the detection of pathogenic bacteria in fecal samples.

Pathogen	Gene	Primers	Sequences	Amplicons (bp)	Annealing Temperature °C	Ref.
*Coxiella* *burnetii*	*IS1111*	Trans-1	TATGTATCCACCGTAGCCAGT	687	64	[50]
		Trans-2	CCCAACAACACCTCCTTATTC			
*Mycobacterium* spp.	16SrDNA	MycogenF	AGAGTTTGATCCTGGCTCAG	1030	62	[51]
		MycogenR	TGCACACAGGCCACAAGGGA			
*Brucella* spp.	*bcsp31*	B4	TGGCTCGGTTGCCAATATCAA	223	60	[52]
		B5	CGCGCTTGCCTTTCAAGGTCTG			
*Francisella* *tularensis*	*TUL4*	TUL4–435	TCGAAGACGATCAGATACCGTCG	400	55	[53]
		TUL4–863	TGCCTTAAACTTCCTTGCGAT			

**Table 3:** Results of bacteriological and molecular analyses conducted on wolf fecal samples.

Sample Number	*Listeria* *monocytogenes*	*Campylobacter* spp.	*Yersinia* spp.	*Salmonella* spp.	STEC	ESBL	CRE	*Coxiella* *burnetii*	*Mycobacterium* spp.	*Brucella* spp.	*Francisella* *tularensis*
1	+	−	+	−	−	−	−	−	−	−	−
2	−	−	+	−	−	−	−	−	−	−	−
3	−	−	−	−	−	−	−	−	−	−	−
4	−	−	−	−	−	−	−	−	−	−	−
5	−	−	−	−	−	−	−	−	−	−	−
6	−	−	+	−	−	−	−	−	−	−	−
7	−	−	+	−	−	−	−	−	−	−	−
8	−	−	−	−	−	−	−	−	−	−	−
9	−	−	−	−	−	−	−	−	−	−	−
10	−	−	−	−	−	−	−	−	−	−	−
11	−	−	−	−	−	−	−	−	−	−	−
12	−	−	−	−	+	−	−	−	−	−	−
13	−	−	−	−	−	+	−	−	−	−	−
14	−	−	−	−	−	−	−	−	−	−	−
15	−	−	−	−	−	−	−	−	−	−	−
16	−	−	−	−	−	+	−	−	−	−	−
Total	1	0	4	0	1	2	0	0	0	0	0

Legend: STEC: Shiga Toxin-Producing *Escherichia coli*; ESBL: Extended Spectrum β-Lactamase-producing Enterobacteriaceae; CRE: carbapenemase-producing Enterobacteriaceae; +: Positive; −: negative.

## Data Availability

The data presented in this study are available within the article.
